# Supplementary material for: Enhanced Viral Activity in the Surface Microlayer of the Arctic and Antarctic Oceans
Source: Microorganisms. 2021 Feb 4;9(2):317. doi: 10.3390/microorganisms9020317 (PMC7913828; doi:10.3390/microorganisms9020317)
Supplement: Supplementary file 1 [file microorganisms-09-00317-s001.pdf]

microorganisms

Supplementary Materials: Enhanced Viral Activity in the Surface Microlayer of the Arctic and Antarctic Oceans

Dolors Vaqué [1,*,†], Julia A. Boras [1,†], Jesús Maria Arrieta [2], Susana Agustí [3], Carlos M. Duarte [3] and Maria Montserrat Sala [1]

Publisher's Note: MDPI stays neutral with regard to jurisdictional claims in published maps and institutional affiliations.

Copyright: © 2021 by the authors. Submitted for possible open access publication under the terms and conditions of the Creative Commons Attribution (CC BY) license (http://creativecommons.org/licenses/by/4.0/).

Table S1. Pearson correlation coefficient among all variables in the surface microlayer (SML) and in the subsurface microlayer (SSW) of the Arctic sea water. Significant values are in bold.

SML	VA	PA	VPR	PHP	VPL	RLC	%VMM	HNF	PNF	Temp	Sal	DOC	PO4	SiO4	NO3/NO2	NH4	Wind	UVB
VA	-	**0.621**	**0.434**	-0.05	-0.01	**0.630**	0.257	0.142	0.241	**0.436**	0.290	**0.798**	**0.567**	**0.703**	0.071	0.355	**-0.512**	0.386
PA	**0.621**		-0.361	0.376	0.089	**0.784**	0.416	0.223	0.224	0.248	**0.480**	**0.633**	**0.485**	**0.690**	**0.422**	0.251	-0.114	0.237
VPR	**0.434**	-0.361		**-0.483**	-0.158	0.325	0.104	-0.091	-0.028	-0.154	-0.279	0.014	0.113	0.073	-0.271	0.124	-0.306	0.254
PHP	-0.05	0.376	**-0.483**		0.012	0.142	0.270	**-0.536**	0.315	0.068	0.035	0.320	-0.092	0.146	0.326	0.355	0.205	-0.004
VPL	-0.01	0.089	-0.158	0.012		-0.032	-0.197	0.071	-0.113	0.240	0.176	-0.119	0.142	-0.052	**0.892**	0.345	0.124	0.318
RLC	**0.630**	**0.784**	0.325	0.142	-0.032		**0.890**	0.178	0.097	0.128	-0.001	**0.568**	0.315	0.504	0.316	0.359	**-0.798**	0.342
%VMM	0.257	0.416	0.104	0.270	-0.197	**0.890**		-0.052	-0.152	-0.322	-0.364	0.245	-0.129	0.171	0.157	0.061	-0.519	0.135
HNF	0.142	0.223	-0.091	**-0.536**	0.071	0.178	-0.052		**-0.578**	0.292	0.001	-0.466	0.329	-0.166	0.059	-0.262	-0.283	-0.202
PNF	0.241	0.224	-0.028	0.315	-0.113	0.097	-0.152	**-0.578**		0.306	0.407	**0.593**	0.404	**0.686**	0.229	0.394	-0.088	0.410
Temp	**0.436**	0.248	-0.154	0.068	0.240	0.128	-0.322	0.292	0.306		**0.593**	**0.442**	**0.767**	**0.435**	0.214	0.303	-0.379	**0.539**
Sal	0.290	**0.480**	-0.279	0.035	0.176	-0.001	-0.364	0.001	0.407	**0.593**		0.390	**0.560**	**0.542**	0.259	0.269	0.059	0.221
DOC	**0.798**	**0.633**	0.014	0.320	-0.119	**0.568**	0.245	-0.466	**0.593**	**0.442**	0.390		0.415	**0.889**	0.335	**0.486**	**-0.471**	0.261
PO4	**0.567**	**0.485**	0.113	-0.092	0.142	0.315	-0.129	0.329	0.404	**0.767**	**0.560**	0.415		**0.632**	0.388	0.345	**-0.531**	0.309
SiO4	**0.703**	**0.690**	0.073	0.146	-0.052	0.504	0.171	-0.166	**0.686**	**0.435**	**0.542**	**0.889**	**0.632**		0.301	0.259	-0.255	0.278
NO3/NO2	0.071	**0.422**	-0.271	0.326	**0.892**	0.316	0.157	0.059	0.229	0.214	0.259	0.335	0.388	0.301		0.266	-0.045	-0.020
NH4	0.355	0.251	0.124	0.355	0.345	0.359	0.061	-0.262	0.394	0.303	0.269	**0.486**	0.345	0.259	0.266	-	-0.076	0.280
Wind	**-0.512**	-0.114	-0.306	0.205	0.124	**-0.798**	-0.519	-0.283	-0.088	-0.379	0.059	**-0.471**	**-0.531**	-0.255	-0.045	-0.076		0.240
UVB	0.386	0.237	0.254	-0.004	0.318	0.342	0.135	-0.202	0.410	**0.539**	0.221	0.261	0.309	0.278	-0.020	0.280	0.240	

SSW	VA	PA	VPR	PHP	VPL	RLC	%VMM	HNF	PNF	Temp	Sal	DOC	PO4	SiO4	NO3/NO2	NH4	Wind	UVB
VA		0.253	**0.562**	0.165	0.217	0.197	0.147	-0.160	0.306	0.269	0.081	0.089	-0.101	0.226	-0.286	0.015	-0.152	0.391
PA	0.253		**-0.626**	0.386	0.459	0.194	-0.214	0.364	0.288	0.300	**0.496**	0.135	0.329	**0.613**	**0.489**	-0.023	-0.031	0.184
VPR	**0.562**	**-0.626**		-0.201	0.147	0.007	0.165	-0.446	-0.002	-0.254	0.395	0.046	-0.365	-0.358	**-0.646**	-0.102	-0.093	0.123
PHP	0.165	0.386	-0.201		-0.070	0.455	**0.709**	-0.098	**0.585**	0.226	0.130	0.218	0.033	0.108	0.274	-0.262	0.304	-0.264

**VPL**	0.217	0.459	0.147	-0.070		**0.701**	-0.076	-0.166	0.426	0.248	0.064	0.231	0.285	0.574	0.474	0.585	-0.441	0.594
**RLC**	0.197	0.194	0.007	0.455	**0.701**		**0.568**	-0.140	0.382	-0.071	-0.210	0.280	-0.402	0.306	0.105	0.057	-0.395	**0.890**
**%VMM**	0.147	-0.214	0.165	**0.709**	-0.076	**0.568**		-0.022	0.064	-0.250	-0.367	0.148	**-0.926**	-0.179	-0.353	-0.538	-0.085	0.661
**HNF**	-0.160	0.364	-0.446	-0.098	-0.166	-0.140	-0.022		**-0.508**	0.316	0.103	-0.373	0.257	-0.180	0.067	0.119	-0.199	-0.090
**PNF**	0.306	0.288	-0.002	**0.585**	0.426	0.382	0.064	**-0.508**		0.228	**0.524**	**0.675**	0.191	**0.731**	**0.546**	-0.239	0.219	**0.586**
**Temp**	0.269	0.300	-0.254	0.226	0.248	-0.071	-0.250	0.316	0.228		**0.761**	0.204	**0.523**	**0.423**	0.124	0.159	-0.285	0.423
**Sal**	0.081	**0.496**	0.395	0.130	0.064	-0.210	-0.367	0.103	**0.524**	**0.761**		0.295	**0.517**	**0.704**	0.354	0.071	0.071	0.242
**DOC**	0.089	0.135	0.046	0.218	0.231	0.280	0.148	-0.373	**0.675**	0.204	0.295		0.115	0.368	0.263	0.263	-0.177	0.418
**PO4**	-0.101	0.329	-0.365	0.033	0.285	-0.402	**-0.926**	0.257	0.191	**0.523**	**0.517**	0.115		0.323	**0.519**	**0.474**	-0.127	-0.144
**SiO4**	0.226	**0.613**	-0.358	0.108	0.574	0.306	-0.179	-0.180	**0.731**	0.423	**0.704**	0.368	0.323		**0.522**	-0.009	0.090	0.460
**NO3/NO2**	-0.286	**0.489**	**-0.646**	0.274	0.474	0.105	-0.353	0.067	**0.546**	0.124	0.354	0.263	**0.519**	**0.522**		0.258	-0.039	-0.052
**NH4**	0.015	-0.023	-0.102	-0.262	0.585	0.057	-0.538	0.119	-0.239	0.159	0.071	-0.038	**0.474**	-0.009	0.258		0.005	0.341
**Wind**	-0.152	-0.031	-0.093	0.304	-0.441	-0.395	-0.085	-0.199	0.219	-0.285	0.071	-0.177	-0.127	0.090	-0.049	0.005		0.360
**UVB**	0.391	0.184	0.123	-0.264	0.594	**0.890**	0.661	-0.090	**0.586**	0.423	0.242	0.418	-0.144	0.460	-0.052	0.341	0.360	

Table S2. Pearson correlation coefficient among all variables in the surface microlayer (SML) and in the subsurface microlayer (SSW) of Antarctic sea water. Significant values are in bold.

**SML**	**VA**	**PA**	**VPR**	**PHP**	**VPL**	**RLC**	**%VMM**	**HNF**	**PNF**	**Temp**	**Sal**	**DOC**	**PO4**	**SiO4**	**NO3/NO2**	**NH4**	**Wind**	**UVB**
**VA**	-	**0.517**	**0.906**	**0.665**	**0.636**	**0.874**	0.289	0.441	0.327	-	-	-	**-0.593**	-0.368	-0.204	0.142	**-0.423**	**-0.627**
**PA**	**0.517**		0.107	0.365	**0.787**	**0.700**	-0.061	0.667	0.692	-	-	-	-0.443	**-0.595**	-0.246	0.075	**-0.492**	-0.399
**VPR**	**0.906**	0.107		**0.592**	0.470	**0.892**	0.484	0.247	0.057	-	-	-	**-0.486**	0.152	-0.089	0.141	-0.249	-0.500
**PHP**	**0.665**	0.365	**0.592**		0.468	**0.970**	0.661	0.388	0.099	-	-	-	**-0.519**	-0.345	**-0.518**	-	-0.284	**-0.607**
**VPL**	**0.636**	**0.787**	0.470	0.468		0.569	-0.023	**0.987**	0.767	-	-	-	-0.422	-0.397	-	0.088	-0.643	-0.703
**RLC**	**0.874**	**0.700**	**0.892**	**0.970**	0.569		0.669	0.466	0.109	-	-	-	-0.420	-0.395	-	-	**-0.815**	-0.599
**%VMM**	0.289	-0.061	0.484	0.661	-0.023	0.669		0.001	-0.543	-	-	-	0.294	0.268	-	-	-0.600	-0.415
**HNF**	0.441	0.667	0.247	0.388	**0.987**	0.466	0.001		**0.801**	-	-	-	-0.299	-0.273	-	-	-0.584	-0.431
**PNF**	0.327	0.692	0.057	0.099	0.767	0.109	-0.543	**0.801**		-	-	-	-0.549	-0.527	-	-	-0.242	0.805

**Temp**	-	-	-	-	-	-	-	-	-		-	-	-	-	-	-	-	-
**Sal**	-	-	-	-	-	-	-	-	-	-		-	-	-	-	-	-	-
**DOC**	-	-	-	-	-	-	-	-	-	-	-		-	-	-	-	-	-
**PO4**	**-0.593**	-0.443	**-0.486**	**-0.519**	-0.422	-0.420	0.294	-0.299	-0.549	-	-	-		-	-	-	0.084	0.110
**SiO4**	-0.368	**-0.595**	0.152	-0.345	-0.397	-0.395	0.268	-0.273	-0.527	-	-	-	**0.860**		-	-	0.063	-0.109
**NO3+NO2**	-0.204	-0.246	-0.089	**-0.518**	-	-	-	-	-	-	-	-	**0.865**	**0.747**		-	-0.045	0.562
**NH4**	0.142	0.075	0.141	-	0.088	-	-	-	-	-	-	-	0.186	-0.004	-0.290		-0.361	-0.506
**Wind**	**-0.423**	**-0.492**	-0.249	-0.284	-0.643	**-0.815**	-0.600	-0.584	-0.242	-	-	-	0.084	0.063	-0.045	-0.361		0.226
**UVB**	**-0.627**	-0.399	-0.500	**-0.607**	-0.703	-0.599	-0.415	-0.431	0.805	-	-	-	0.110	-0.109	0.562	-0.506	0.226	

**SSW**	**VA**	**PA**	**VPR**	**PHP**	**VPL**	**RLC**	**%VMM**	**HNF**	**PNF**	**Temp**	**Sal**	**DOC**	**PO4**	**SiO4**	**NO3+NO2**	**NH4**	**Wind**	**UVB**
**VA**		0.309	**0.934**	0.329	0.342	0.241	-0.167	0.487	-0.009	-0.043	**-0.505**	**0.542**	-0.377	-0.129	-0.003	0.093	**-0.508**	**-0.704**
**PA**	0.309		0.024	**0.577**	0.512	0.392	-0.021	0.244	-0.224	**0.445**	0.240	-0.141	-0.227	-0.328	-0.009	-0.391	-0.369	-0.222
**VPR**	**0.934**	0.024		0.133	0.272	0.180	-0.219	0.569	0.070	-0.217	**-0.645**	**0.647**	-0.320	-0.014	0.040	0.284	**-0.406**	**-0.715**
**PHP**	0.329	**0.577**	0.133		-0.119	-0.250	-0.481	**0.932**	0.608	0.044	-0.224	0.291	-0.140	-0.289	-0.174	0.283	0.000	-0.517
**VPL**	0.342	0.512	0.272	-0.119		0.313	0.111	-0.210	-0.264	-0.420	0.229	0.282	-0.350	-0.215	0.718	0.835	-0.098	0.506
**RLC**	0.241	0.392	0.180	-0.250	0.313		**0.912**	-0.581	**0.898**	-0.091	-0.066	0.422	-0.307	-0.238	0.142	-0.133	0.339	0.656
**%VMM**	-0.167	-0.021	-0.219	-0.481	0.111	**0.912**		-0.672	**-0.867**	-0.092	-0.171	0.104	-0.058	-0.100	-0.059	0.107	0.545	0.787
**HNF**	0.487	0.244	0.569	**0.932**	-0.210	-0.581	-0.672		**0.811**	-0.194	-0.197	0.499	-0.524	-0.400	-0.179	0.342	**-0.918**	-0.842
**PNF**	-0.009	-0.224	0.070	0.608	-0.264	**0.898**	**-0.867**	**0.811**		-0.210	-0.197	-0.030	-0.119	-0.151	-0.253	0.318	-0.700	-0.626
**Temp**	-0.043	**0.445**	-0.217	0.044	-0.420	-0.091	-0.092	-0.194	-0.210		0.336	-0.241	**0.425**	0.369	0.419	0.021	-0.070	-0.417
**Sal**	**-0.505**	0.240	**-0.645**	-0.224	0.229	-0.066	-0.171	-0.197	-0.197	0.336		**-0.697**	0.126	0.106	-0.013	**-0.874**	0.270	0.261
**DOC**	**0.542**	-0.141	**0.647**	0.291	0.282	0.422	0.104	0.499	-0.030	-0.241	**-0.697**		-0.112	0.097	0.079	-0.189	-0.045	-0.231
**PO4**	-0.377	-0.227	-0.320	-0.140	-0.350	-0.307	-0.058	-0.524	-0.119	**0.425**	0.126	-0.112		**0.781**	**0.935**	0.065	**0.400**	0.094
**SiO4**	-0.129	-0.328	-0.014	-0.289	-0.215	-0.238	-0.100	-0.400	-0.151	0.369	0.106	0.097	**0.781**		**0.784**	-0.167	0.312	-0.226
**NO3+NO2**	-0.003	-0.009	0.040	-0.174	0.718	0.142	-0.059	-0.179	-0.253	0.419	-0.013	0.079	**0.935**	**0.784**		-0.050	0.242	0.129
**NH4**	0.093	-0.391	0.284	0.283	-0.835	-0.133	0.107	0.342	0.318	0.021	**-0.874**	-0.189	0.065	-0.167	-0.050		-0.094	-0.296
**Wind**	**-0.508**	-0.369	**-0.406**	0.000	-0.098	0.339	0.545	**-0.918**	-0.700	-0.070	0.270	-0.045	**0.400**	0.312	0.242	0.094		0.226

**UVB**	**-0.704**	-0.222	**-0.715**	-0.517	0.506	0.656	0.787	-0.842	-0.626	-0.417	0.261	-0.231	0.094	-0.226	0.129	-0.296	0.226
